# Increased Intrathecal Chemokine Receptor CCR2 Expression in Multiple Sclerosis

**Published:** 2007-12-18

**Authors:** Hideto Nakajima, Masakazu Sugino, Fumiharu Kimura, Toshiaki Hanafusa, Toshiyuki Ikemoto, Akira Shimizu

**Affiliations:** 1 Division of Neurology, Department of Internal Medicine I, Osaka Medical College; 2 Department of Internal Medicine, Seikeikai Hospital, Sakai, Japan; 3 Department of Central Laboratory, Osaka Medical College, Takatsuki, Japan

**Keywords:** multiple sclerosis, CCR2, CXCR3, CCR4, chemokine receptor, chemokine

## Abstract

Expression of CCR2, CXCR3 and CCR4 on CD4^+^ T or CD8^+^ T cells in blood and cerebrospinal fluid (CSF) for multiple sclerosis (MS) was measured by 3-color flow cytometry, and compared to blood from healthy controls and CSF from patients with other inflammatory neurological diseases (INDs). CD4^+^CXCR3^+^/CD4^+^CCR4^+^ ratio (representing Th1/Th2 balance) was higher in both CSF and blood of MS patients than those of IND patients or healthy controls. Percentage of CCR2-positive T cells was significantly higher in CSF from MS patients. Increased CCR2 expression on T cells in CSF and Th1/Th2 imbalance may reflect the pathological processes involved in MS.

## Introduction

Multiple sclerosis (MS) is a chronic and progressive inflammatory immune-mediated demyelinating disease. Recruitment of peripheral blood mononuclear cells into the central nervous system (CNS) plays a crucial role in the pathogenesis of this condition. Chemokines are low molecular weight cytokines produced in inflamed tissue, mediating the recruitment of specific leukocyte populations expressing chemokine receptors. Recent studies have shown that T cells expressing CCR5 and CXCR3 can be detected within perivascular lesions of brains with MS [Simpson et al. 1998; Balashov et al. 1999; Sorensen et al. 1999], and T cells expressing these receptors are increased in cerebrospinal fluid (CSF) compared with peripheral blood [Kivisakk et al. 2002; Sorensen et al. 1999, 2002; Teleshova et al. 2002]. Increased expression of CXCR3 on CD4^+^ T cells in peripheral blood from MS patients compared to those of healthy individuals has also been reported [Nakajima et al. 2004a]. Since such chemokine receptors are preferentially expressed on Th1 cells, these cells are considered critical in the pathogenesis of MS. In contrast, expression of CCR4 and CCR3 (Th2-type receptors) on CD4^+^ T cells is significantly decreased in the blood of MS patients at the time of relapse compared to healthy controls, and CCR4 expression is significantly decreased in CSF CD4^+^ T cells compared to peripheral blood CD4^+^ T cells [Misu et al. 2001]. These findings suggest that Th2 response is suppressed in the acute phase of MS. Th1/Th2 balance may thus play an important role in this relapsing-remitting disorder [Misu et al. 2001; Nakajima et al. 2004a].

In experimental autoimmune encephalomyelitis (EAE), CCL2 and CCR2, the main receptor for CCL2, are expressed on astrocytes, macrophages and T cells in CNS lesions during the acute phase [Jee et al. 2002]. CCR2-knockout mice are also resistant to EAE [Fife et al. 2000; Izikson et al. 2000]. These findings suggest that CCR2 and CCL2 are key susceptibility factors in EAE. Unexpectedly, CCL2 concentrations in CSF from MS patients after relapse are reduced when concentrations of other inflammatory chemokines such as CCL1, CCL5 and CXCL10 are increased [Bartosik-Psujek and Stelmasiak, 2005; Mahad et al. 2002; Nakajima et al. 2004a; Sorensen et al. 1999, 2001]. Since CCL2 has been shown to induce Th2 reactions [Gu et al. 2000; Nakajima et al. 2001], CCL2 production may be suppressed in the Th1-predominant immunological conditions of active MS. However, both CCR2 and CCL2 are abundantly detected in plaque lesions from MS patients [Mahad and Randsohoff, 2003; McManus et al. 1998]. Multiple compensatory mechanisms, particularly concerned with CCL2-CCR2, appear to exist in the pathogenesis of MS.

The present study examined expressions of CXCR3, CCR4 and CCR2 on CD4^+^ T and CD8^+^ T cells in blood and CSF from relapsing-remitting MS patients and CSF from patients with other inflammatory neurological diseases (INDs). Comparing these groups, we evaluated specific T-cell populations trafficking to the MS lesion. The results indicate that CCR2 upregulation in CSF occurs in MS.

## Materials and Methods

### Subjects

Paired blood and CSF samples were obtained from 10 patients with relapsing-remitting MS during the relapse phase (8 women, 2 men; mean (±SD) age, 44 ± 9 years (range, 20–56 years); mean disease duration 6 ± 5 years (range, 0.5–13 years); mean expanded disability status scale (EDSS) [Kurtzke et al. 1983] 3 ± 1.5 (range, 1.5–5.5)) (Table 1). All patients included in this study displayed definite MS, according to McDonald criteria [McDonald et al. 2001]. Samples were obtained from these patients before administration of corticosteroid pulse therapy. CSF samples were also collected from 10 patients with IND (6 women, 4 men; mean age, 39 ± 16 years (range, 18–57 years)). IND comprised viral meningitis/encephalitis (n = 8), acute disseminated encephalomyelitis (n = 1) or neuro-Behçet’s disease (n = 1). All 10 patients displayed mononuclear pleocytosis (Table 1). As control samples, blood samples were collected from 10 healthy individuals (4 women, 6 men; mean age, 33 ± 9 years (range, 21–52 years)).

### Flow Cytometry

Venous blood was collected in heparinized tubes and analyzed within 2 h after sampling. CSF cells were collected by centrifugation (250 × *g* for 10 min) and resuspended in PBS with 1% BSA. Whole blood and CSF cells (≥5 × 10^3^ cells/test) were labeled with directly conjugated monoclonal antibodies, according to the instructions of the manufacturer, using anti-CD3 PerCP (Becton Dickinson, San Jose, CA), anti-CD4 FITC, anti-CD8 FITC (Pharmingen, San Diego, CA), anti-CCR4 PE, anti-CXCR3 PE (Pharmingen, San Diego, CA) and anti-CCR2 PE (Dako, Kyoto, Japan), in addition to isotype-specific antibody controls. Cells were fixed in 2% paraformaldehyde and stored in the dark before analysis using a FACS flow cytometer (Becton Dickinson). Flow cytometry data were processed using CellQuest software (Becton Dickinson). Data are reported as percentages of all T cells (identified as CD3^+^ cells) staining positively for CD4^+^CXCR3^+^, CD8^+^CXCR3^+^, CD4^+^CCR4^+^, CD8^+^CCR4^+^, CD4^+^CCR2^+^ or CD8^+^CCR2^+^.

### Statistical analysis

Comparisons between expressions of chemokine receptors in CSF and blood from MS patients, CSF from patients with IND and blood from healthy controls were performed by nonparametric Mann-Whitney U and Kruskall-Wallis tests. Values of p < 0.05 were considered statistically significant.

## Results

### Comparison of Th1/Th2-related chemokine receptor expression

**Figure 1:**
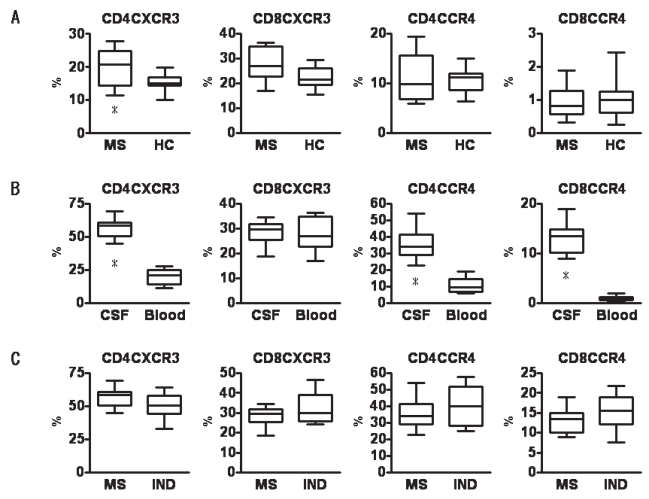
Comparison of chemokine receptor expression on blood T cells between MS and healthy controls (**A**), on T cells between CSF and blood from MS (**B**), and on CSF T cells between MS and IND (**C**). Data are shown as percentages of all T cells staining positive for CD4^+^CXCR3^+^, CD8^+^CXCR3^+^, CD4^+^CCR4^+^ or CD8^+^CCR4^+^.

The percentage of CD4^+^ or CD8^+^ T cells expressing these chemokine receptors among all CD3^+^ T cells was determined by 3-color flow cytometry using anti-CD3 antibody and anti-CD4 antibody or anti-CD8 antibody and one of the following anti-chemokine receptor antibodies: anti-CXCR3 antibody; anti-CCR4 antibody; or anti-CCR2 antibody. In peripheral blood, percentages of CD4^+^CXCR3^+^ cells were higher for MS patients than for healthy controls (p = 0.013; Fig. 1A). However, no differences were observed in percentages of other subsets (Fig. 1A). When comparing CSF and blood in MS patients, percentages of all subsets other than CD8^+^CXCR3^+^ cells were significantly higher in CSF (Fig. 1B). Expressions of all chemokine receptors were also elevated in CSF from IND patients compared levels in blood for healthy controls (Fig. 1C). However, no differences were observed between percentages of CD4^+^CXCR3^+^, CD8^+^CXCR3^+^, CD4^+^CCR4^+^ or CD8^+^CCR4^+^ cells between groups (Fig. 1C). These findings suggest that activated T lymphocytes cross the blood-brain barrier (BBB) and contribute to inflammatory response in the pathogenesis of MS.

### Comparison of CD4^+^CXCR3^+^/CD4^+^CCR4^+^ ratios

**Figure 2:**
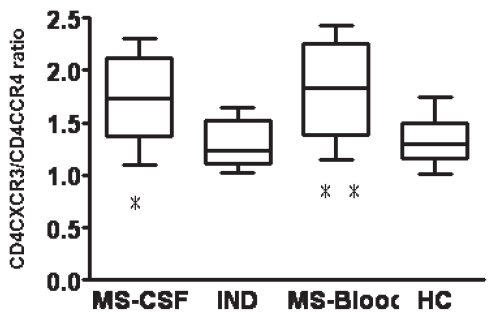
Comparison of CD4^+^CXCR3^+^/CD4^+^CCR4^+^ between CSF and blood from MS patients, CSF from patients with IND, and blood from healthy controls. As CD4^+^CXCR3^+^ cells are Th1 cells and CD4^+^CCR4^+^ cells are Th2 cells, CD4^+^CXCR3^+^/CD4^+^CCR4^+^ ratio represents Th1/Th2 balance.

As CXCR3 is expressed on Th1 cells and CCR4 is expressed on Th2 cells, CD4^+^CXCR3^+^/CD4^+^CCR4^+^ ratio represents Th1/Th2 balance. CD4^+^CXCR3^+^/CD4^+^CCR4^+^ ratio in blood was significantly higher for MS patients than for healthy controls (p = 0.034). In MS patients, CD4^+^CXCR3^+^/CD4^+^CCR4^+^ ratio was similar between CSF and blood. However, CD4^+^CXCR3^+^/CD4^+^CCR4^+^ ratio in CSF was significantly lower for IND patients than for MS patients (p = 0.042; Fig. 2). These findings suggest that Th1/Th2 imbalance indicates the pathological nature of MS.

### Comparison of CCR2 expression

**Figure 3:**
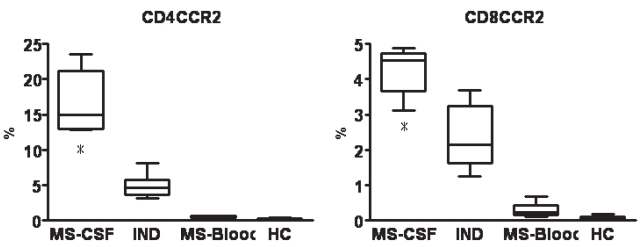
Comparison of CCR2 expression on CD4^+^ and CD8^+^ T cells between CSF and blood from MS patients, CSF from patients with IND, and blood from healthy controls. Data are shown as percentages of all T cells staining positive for CD4^+^CCR2^+^or CD8^+^CCR2^+^.

When comparing CSF and blood in MS patients, percentages of CD4^+^CCR2^+^ and CD8^+^CCR2^+^ cells were significantly increased in CSF (p = 0.002 and 0.014, respectively; Fig. 3). In CSF, CCR2 expression on CSF CD4^+^ and CD8^+^ cells was also higher for MS patients than for IND patients (p = 0.023 and 0.028, respectively; Fig. 3). CCR2 expression was highest in CSF from MS patients (Fig. 3). These findings suggest that CCR2-expressing T cells play a pivotal role in the pathogenesis of MS.

## Discussion

Recruitment of autoreactive T lymphocytes from blood to the CNS plays a crucial role in the pathogenesis of MS, as this process initiates the inflammatory response leading to demyelination and axonal degeneration. Interactions between chemokines and chemokine receptors are thought to be important in the activation and migration of activated T lymphocytes across the BBB. Investigation of chemokine receptor expression on T cells in the blood and CSF of MS patients is thus key to understanding the pathogenic processes in MS. In the present study, percentages of CXCR3-, CCR4- and CCR2-expressing T cells were higher in CSF than in blood, and this characteristic was more pronounced in CD4 T cells than in CD8 T cells. These findings suggest that activated T lymphocytes, including myelin-reactive CD4^+^ T cells, cross the BBB and contribute to the inflammatory response (Fig. 1). No differences in percentages of CXCR3-expressing CD8^+^ T cells were identified in this study between CSF and blood from MS patients. Previously, a significant increase in percentage of CXCR3-expressing CD8^+^ T cells was found in CSF compared to blood from MS patients [Misu et al. 2001]. In the present study, expression of chemokine receptors was measured using 3-color flow cytometry. Expressions of each chemokine on CD4^+^ and CD8^+^ T cells are shown as percentages of all T cells. Since our results demonstrated a greatly increased percentage of CXCR3-expressing CD4^+^ T cells in CSF from MS patients, the percentage of CXCR3-expressing CD8^+^ T cells in CSF was relatively low, as in blood. Technical approaches to the detection of chemokine receptors by FACS analysis strongly affect the results [Kivisakk et al. 2002].

No difference in CD4^+^CXCR3^+^/CD4^+^CCR4^+^ ratio was seen between CSF and blood from MS patients (Fig. 2). Previous studies comparing blood from MS patients and healthy individuals have reported elevations of Th1-type chemokine receptor CXCR3 and CCR5 expression and reductions in the expression of Th2-type chemokine receptors CCR4 and CCR3 in patients with MS [Misu et al. 2001; Nakajima et al. 2004; Sorensen et al. 1999]. Those findings suggest that Th1 predominance in the blood and CSF is involved in the pathogenesis of MS. Our study shows that activated T cells expressing chemokine receptors invade the CNS of IND patients, as seen in MS patients. However, CD4^+^CXCR3^+^/CD4^+^CCR4^+^ ratio in the CSF was significantly higher for MS patients than for IND patients (Fig. 2). Th1/Th2 imbalance may thus reflect the pathological nature of MS and indicate the degree of CNS inflammation.

Our previous study showed that expressions of CCR2 and CD14 on monocytes in the blood of MS patients are markedly decreased, and revealed a significant negative correlation between CD4^+^CXCR3^+^/CD4^+^CCR4^+^ ratio and CCR2 and CD14 expressions on monocytes [Nakajima et al. 2004b]. To elucidate whether decreases in percentage of CD14^+^CCR2^+^ monocytes in blood corresponded to those in CSF, the present study investigated CCR2-expressing monocytes in the CSF of MS patients. No CD14^+^CCR2^+^ monocytes were detected in the CSF of patients. However, the percentage of CCR2-expressing T cells was markedly increased in the CSF of MS patients. The percentage of these cells among blood T cells was extremely low. Although CCR2-expressing T cells were detectable in CSF from patients with IND, CCR2 expression was clearly decreased compared to MS patients (Fig. 1C). CCL2 and CCR2 are thought to play pivotal roles in the development and relapse of MS and EAE. However, the immune cascade, including CCL2 and CCR2, in the CNS is poorly understood. CCL2 and CCR2 expressions in the brain are closely associated with disease activity in EAE [Jee et al. 2002]. CCL-knockout mice are resistant to EAE and show significant reductions in CNS macrophage invasion [Huang et al. 2001]. Although T cells from CCL2 knockout mice transfer EAE to wild-type mice, wild-type T cells fail to cause clinical EAE in CCL-knockout mice [Huang et al. 2001]. CCR2-knockout mice likewise display decreased susceptibility to EAE [Fife et al. 2000; Izikson et al. 2000]. T cells from CCR2 knockout mice are able to induce EAE in wild-type mice, whereas CCR2-knockout recipients of wild-type T cells fail to develop EAE [Fife et al. 2000]. These findings suggest that CCL2/CCR2-induced macrophage recruitment is critical in the pathogenesis of EAE. CCL2 and CCR2 are abundantly detected in plaque lesions of MS patients [Mahad and Ransohoff, 2003; McManus et al. 1998]. An increased number of intrathecal CCR2-expressing T cells was also demonstrated in the present study. These cells may thus play a pathogenic role in acute lesions of MS.

Recent studies have shown that CCL2 concentrations in CSF and serum from early active MS patients are reduced, but increase during remission [Bartosik-Psujek and Stelmasiak, 2005; Mahad et al. 2002; Nakajima et al. 2004a; Sorensen et al. 1999, 2001, 2004]. Reduced CCL2 levels have also been seen in clinical isolated syndrome [Sorensen et al. 1999]. In contrast, elevated levels of CCL2 are present in viral meningitis and CNS vasculitis [Mahad et al. 2002] and in HIV-associated dementia [Franciotta et al. 2001]. This reduced CCL2 expression is a key to elucidating the pathogenesis of MS. As a mechanism for the low CCL2 levels in CSF, Mahad et al. suggested that CCL2 was consumed by circulating CCR2-expressing mononuclear cells, and that CCR2 was downregulated on cells migrating in response to CCL2 via the in vitro BBB model [Mahad et al. 2006]. In the present study, CCR2-expressing monocytes were not detected in CSF. This downregulation of CCR2 on monocytes in CSF would be due to internalization of CCR2 following binding of CCL2 during transmigration to the CSF. Conversely, percentages of CCR2-expressing T cells were elevated in CSF of MS patients. Since a large amount of CCR2-expressing T cells are thought to transmigrate to the CSF in MS, these cells would be detected even after consumption of CCL2. Increases in numbers of CCR2-expressing T cells in CSF are thought to be characteristic for MS, and these cells may be associated with the relapsing-remitting autoimmune reactions of MS.

## Figures and Tables

**Table 1:** Patient demographics and CSF findings.

	Gender (M:F)	Age median (range) (years)	CSF WBC (/mm3)	CSF protein (mg/dl)
MS (n = 10)	2:8	44 (20–56)	20 ± 6	53 ± 11
IND (n = 10)	4:6	39 (18–57)	183 ± 112	62 ± 24
Healthy (n = 10)	6:4	33 (21–52)	NA	NA

CSF WBC and protein are presented as mean ± SD.

**Abbreviation:** CSF: cerebrospinal fluid; WBC: white blood cells; MS: multiple sclerosis; IND: other inflammatory neurological diseases; NA: not applicable.

## References

[b1-bmi-2007-463] Balashov KE Rottman JB Weiner HL Hancock WW 1999 CCR5(+) and CXCR3(+) T cells are increased in multiple sclerosis and their ligands MIP-1alpha and IP-10 are expressed in demyelinating brain lesions Proc Natl Acad Sci, USA 96 6873 6878 10359806 10.1073/pnas.96.12.6873 PMC22009

[b2-bmi-2007-463] Bartosik-Psujek H Stelmasiak Z 2005 The levels of chemokines CXCL8, CCL2 and CCL5 in multiple sclerosis patients are linked to the activity of the disease Eur J Neurol 12 49 54 15613147 10.1111/j.1468-1331.2004.00951.x

[b3-bmi-2007-463] Fife BT Huffnagle GB Kuziel WA Karpus WJ 2000 CC chemokine receptor 2 is critical for induction of experimental autoimmune encephalomyelitis J Exp Med 192 899 905 10993920 10.1084/jem.192.6.899 PMC2193286

[b4-bmi-2007-463] Franciotta D Martino G Zardini E Furlan R Bergamaschi R Andreoni L Cosi V 2001 Serum and CSF levels of MCP-1 and IP-10 in multiple sclerosis patients with acute and stable disease and undergoing immunomodulatory therapies J Neuroimmunol 115 192 198 11282170 10.1016/s0165-5728(01)00261-2

[b5-bmi-2007-463] Gu L Tseng S Horner RM Tam C Loda M Rollins BJ 2000 Control of TH2 polarization by the chemokine monocyte chemoattractant protein-1 Nature 404 407 411 10746730 10.1038/35006097

[b6-bmi-2007-463] Huang DR Wang J Kivisakk P Rollins BJ Ransohoff RM 2001 Absence of monocyte chemoattractant protein 1 in mice leads to decreased local macrophage recruitment and antigen-specific T helper cell type 1 immune response in experimental autoimmune encephalomyelitis J Exp Med 193 713 726 11257138 10.1084/jem.193.6.713 PMC2193420

[b7-bmi-2007-463] Izikson L Klein RS Charo IF Weiner HL Luster AD 2000 Resistance to experimental autoimmune encephalomyelitis in mice lacking the CC chemokine receptor (CCR)2 J Exp Med 192 1075 1080 11015448 10.1084/jem.192.7.1075 PMC2193310

[b8-bmi-2007-463] Jee Y Yoon WK Okura Y Tanuma N Matsumoto Y 2002 Upregulation of monocyte chemotactic protein-1 and CC chemokine receptor 2 in the central nervous system is closely associated with relapse of autoimmune encephalomyelitis in Lewis rats J Neuroimmunol 128 49 57 12098510 10.1016/s0165-5728(02)00147-9

[b9-bmi-2007-463] Kivisakk P Trebst C Liu Z Tucky BH Sorensen TL Rudick RA Mack M Ransohoff RM 2002 T-cells in the cerebrospinal fluid express a similar repertoire of inflammatory chemokine receptors in the absence or presence of CNS inflammation: implications for CNS trafficking Clin Exp Immunol 129 510 518 12197893 10.1046/j.1365-2249.2002.01947.x PMC1906480

[b10-bmi-2007-463] Kivisakk P Liu Z Trebst C Tucky B Wu L Stine J Mack M Rudick RA Campbell JJ Ransohoff RM 2003 Flow cytometric analysis of chemokine receptor expression on cerebrospinal fluid leukocytes Methods 29 319 325 12725798 10.1016/s1046-2023(02)00355-9

[b11-bmi-2007-463] Kurtzke JF 1983 Rating neurologic impairment in multiple sclerosis: an expanded disability status scale (EDSS) Neurology 33 1444 1452 6685237 10.1212/wnl.33.11.1444

[b12-bmi-2007-463] Mahad DJ Howell SJ Woodroofe MN 2002 Expression of chemokines in the CSF and correlation with clinical disease activity in patients with multiple sclerosis J Neurol Neurosurg Psychiatry 72 498 502 11909910 10.1136/jnnp.72.4.498 PMC1737819

[b13-bmi-2007-463] Mahad DJ Ransohoff RM 2003 The role of MCP-1 (CCL2) and CCR2 in multiple sclerosis and experimental autoimmune encephalomyelitis (EAE) Semin Immunol 15 23 32 12495638 10.1016/s1044-5323(02)00125-2

[b14-bmi-2007-463] Mahad D Callahan MK Williams KA Ubogu EE Kivisakk P Tucky B Kidd G Kingsbury GA Chang A Fox RJ Mack M Sniderman MB Ravid R Staugaitis SM Stins MF Ransohoff RM 2006 Modulating CCR2 and CCL2 at the blood-brain barrier: relevance for multiple sclerosis pathogenesis Brain 129 212 223 16230319 10.1093/brain/awh655

[b15-bmi-2007-463] McDonald WI Compston A Edan G Goodkin D Hartung HP Lublin FD McFarland HF Paty DW Polman CH Reingold SC Sandberg-Wollheim M Sibley W Thompson A van den Noort S Weinshenker BY Wolinsky JS 2001 Recommended diagnostic criteria for multiple sclerosis: guidelines from the International Panel on the diagnosis of multiple sclerosis Ann Neurol 50 121 127 11456302 10.1002/ana.1032

[b16-bmi-2007-463] McManus C Berman JW Brett FM Staunton H Farrell M Brosnan CF 1998 MCP-1, MCP-2 and MCP-3 expression in multiple sclerosis lesions: an immunohistochemical and in situ hybridization study J Neuroimmunol 86 20 29 9655469 10.1016/s0165-5728(98)00002-2

[b17-bmi-2007-463] Misu T Onodera H Fujihara K Matsushima K Yoshie O Okita N Takase S Itoyama Y 2001 Chemokine receptor expression on T cells in blood and cerebrospinal fluid at relapse and remission of multiple sclerosis: imbalance of Th1/Th2-associated chemokine signaling J Neuroimmunol 114 207 212 11240033 10.1016/s0165-5728(00)00456-2

[b18-bmi-2007-463] Nakajima H Kobayashi M Pollard RB Suzuki F 2001 Monocyte chemoattractant protein-1 enhances HSV-induced encephalomyelitis by stimulating Th2 responses J Leukoc Biol 70 374 380 11527986

[b19-bmi-2007-463] Nakajima H Fukuda K Doi Y Sugino M Kimura F Hanafusa T Ikemoto T Shimizu A 2004a Expression of TH1/TH2-related chemokine receptors on peripheral T cells and correlation with clinical disease activity in patients with multiple sclerosis Eur Neurol 52 162 168 15528917 10.1159/000081856

[b20-bmi-2007-463] Nakajima H Sugino M Kimura F Hanafusa T Ikemoto T Shimizu A 2004b Decreased CD14+CCR2+ monocytes in active multiple sclerosis Neurosci Lett 363 187 189 15172112 10.1016/j.neulet.2004.04.006

[b21-bmi-2007-463] Simpson JE Newcombe J Cuzner ML Woodroofe MN 1998 Expression of monocyte chemoattractant protein-1 and other beta-chemokines by resident glia and inflammatory cells in multiple sclerosis lesions J Neuroimmunol 84 238 249 9628469 10.1016/s0165-5728(97)00208-7

[b22-bmi-2007-463] Sorensen TL Tani M Jensen J Pierce V Lucchinetti C Folcik VA Qin S Rottman J Sellebjerg F Strieter RM Frederiksen JL Ransohoff RM 1999 Expression of specific chemokines and chemokine receptors in the central nervous system of multiple sclerosis patients J Clin Invest 103 807 815 10079101 10.1172/JCI5150 PMC408141

[b23-bmi-2007-463] Sorensen TL Sellebjerg F Jensen CV Strieter RM Ransohoff RM 2001 Chemokines CXCL10 and CCL2: differential involvement in intrathecal inflammation in multiple sclerosis Eur J Neurol 8 665 672 11784351 10.1046/j.1468-1331.2001.00327.x

[b24-bmi-2007-463] Sorensen TL Ransohoff RM Strieter RM Sellebjerg F 2004 Chemokine CCL2 and chemokine receptor CCR2 in early active multiple sclerosis Eur J Neurol 11 445 449 15257681 10.1111/j.1468-1331.2004.00796.x

[b25-bmi-2007-463] Teleshova N Pashenkov M Huang YM Soderstrom M Kivisakk P Kostulas V Haglund M Link H 2002 Multiple sclerosis and optic neuritis: CCR5 and CXCR3 expressing T cells are augmented in blood and cerebrospinal fluid J Neurol 249 723 729 12111306 10.1007/s00415-002-0699-z

